# Bevacizumab and ranibizumab for neovascular age-related macular degeneration: an updated meta-analysis of randomised clinical trials

**DOI:** 10.1007/s00417-014-2764-6

**Published:** 2014-08-22

**Authors:** Laurent Kodjikian, Evelyne Decullier, Eric H. Souied, Jean-François Girmens, Emilie E. Durand, François R. Chapuis, Laure Huot

**Affiliations:** 1 Service d’ophtalmologie, Hospices Civils de Lyon, Groupement Hospitalier Nord, Hôpital de la Croix-Rousse, 103 Grande Rue de la Croix-Rousse, F-69317 Lyon Cedex 04, France; 2 Université de Lyon, Lyon, F-69007 France; 3 CNRS UMR 5510 Mateis, Villeurbanne, F-69621 France; 4 Pôle Information Médicale Evaluation Recherche, Unité de recherche clinique, Hospices Civils de Lyon, Lyon, F-69003 France; 5 Université Lyon 1, EAM 4128 Santé Individu Société, Lyon, F-69003 France; 6 Service d’ophtalmologie, Centre Hospitalier Intercommunal de Créteil, Créteil, F-94000 France; 7 CRC, Université Paris Est, Créteil, F-94000 France; 8 Centre Hospitalier d’Ophtalmologie des Quinze-Vingts, INSERM-DHOS CIC503, Paris, F-75012 France

**Keywords:** Bevacizumab, Ranibizumab, Neovascular age-related macular degeneration, Meta-analysis

## Abstract

**Purpose:**

Neovascular age-related macular degeneration (AMD) is the main cause of central vision loss among individuals aged 50 years or older in developed countries. The aim of this study was to review systematically the effect of bevacizumab compared to ranibizumab in patients with AMD at 1 year.

**Methods:**

A systematic review was performed on Medline, Embase, and the Cochrane Library and Trial registers to October 2013. Eligibility criteria for selecting studies were randomised controlled trials (RCT) comparing bevacizumab with ranibizumab in patients with neovascular AMD. Odds ratio (OR) and mean difference (MD) estimates were synthesized under fixed- and random-effects models. Heterogeneity was assessed using the Q statistic and I^2^.

**Results:**

Five RCTs were included, representing 2,686 randomised patients. The meta-analysis confirmed the non-inferiority of bevacizumab compared to ranibizumab for change in visual acuity at 1 year (MD 0.57 letters, −1.80 to 0.66, *p* = 0.37, I^2^ = 0 %). Better anatomical results were found for ranibizumab. Bevacizumab was associated with a 34 % increase in the number of patients with at least one serious systemic adverse event (OR 1.34, 1.08 to 1.66, *p* = 0.01, I^2^ = 0 %).

**Conclusions:**

The pooled evidence confirmed that, compared with ranibizumab, bevacizumab was associated with equivalent effects on visual acuity at 1 year and with a higher risk of systemic serious adverse events. The current available data do not show which types of adverse events occur more frequently. In practice, bevacizumab should be used under a risk-management plan until further studies have been carried out to assess accurately the increased risk of systemic adverse events.

## Introduction

Ranibizumab, an anti-vascular endothelial growth factor (anti-VEGF) monoclonal antibody fragment, was developed specifically to treat age-related macular degeneration (AMD) and was approved by the Food and Drug Administration (FDA) and the European Agency for the Evaluation of Medicinal Products (EMEA) in 2006 and 2007, respectively. Nevertheless, bevacizumab, a full anti-VEGF antibody derived from the same parent antibody as ranibizumab, is currently widely used off-label for AMD treatment [1].

Head-to-head studies have been performed in various countries to establish the relative efficacy of bevacizumab versus ranibizumab. The largest study, CATT (Comparison of AMD Treatments Trials), demonstrated the non-inferiority of bevacizumab versus ranibizumab on visual acuity at 1 year [2]. In this study, serious systemic adverse events occurred more frequently in the bevacizumab group, but this was not observed in the other randomised trials [3–5].

In 2012, two meta-analyses including studies with a follow-up period of 1 year were published [3, 6]. Efficacy was studied in only one of them. Chakravarthy et al. confirmed the functional equivalence of the two drugs (visual acuity) [3]. Retinal thickness at fovea was the only anatomical efficacy criterion analysed, with more favourable results for ranibizumab. The safety profiles for intravitreal injections of bevacizumab and ranibizumab appeared to be similar. Although slightly more systemic serious adverse events were observed with bevacizumab, there were no differences between the two drugs for arterial thromboembolic events, non-ocular haemorrhage or death [3, 6].

Since the publication of the last meta-analysis, two further head-to-head trials have been completed [5, 4]. We, therefore, decided to carry out a systematic review and meta-analysis in order to update the results on the functional and anatomical efficacy and safety profile at 1 year of bevacizumab compared with ranibizumab in patients with neovascular AMD.

## Methods

The Cochrane Collaboration methods were used to perform the systematic review [7]. The meta-analysis was performed according to a protocol established before the start of the literature search and data analysis. The study was conducted and reported according to the preferred reporting items for systematic reviews and meta-analyses (PRISMA) checklist [8].

### Eligibility criteria for considering studies for this review

To be eligible for inclusion, trials had to (1) compare the efficacy and/or the safety of bevacizumab and ranibizumab at 1 year (whatever the injection regimen); (2) include only patients with AMD.

### Search methods for identifying studies

The systematic search was performed on Medline (from inception to October 2013), Embase (from January 2000 to October 2013), and the Cochrane Central Register of Controlled Trials (October 2013) using relevant text words and medical subject headings that included all spellings of “macular degeneration” and “bevacizumab” (Box).

**Box: Trial search query**

(bevacizumab[Substance Name] OR bevacizumab[TIAB] OR Avastin[TIAB])

AND

(ranibizumab[Substance Name] OR ranibizumab[TIAB] OR Lucentis[TIAB])

AND

(Macular Degeneration[MeSH Terms] OR Macular Degeneration[TIAB] OR Macular Degenerations[TIAB] OR Age-Related Maculopathies[TIAB] OR Age Related Maculopathies[TIAB] OR Macular Dystrophy[TIAB] OR Macular Dystrophies[TIAB] OR Age-Related Macular Degeneration[TIAB] OR Age Related Macular Degeneration[TIAB] OR Age-Related Macular Degenerations[TIAB] OR Age-Related Maculopathy[TIAB] OR Age Related Maculopathy[TIAB])

AND

(“randomized controlled trial”[PT] OR “controlled clinical trial[PT]” OR “randomized controlled trials”[MeSH Terms] OR “random allocation”[MeSH Terms] OR “double blind method”[MeSH Terms] OR “single-blind method”[MeSH Terms] NOT (animal NOT human)[MeSH Terms] OR “clinical trial”[PT] OR “clin* trial*”[TIAB] OR “placebos”[MeSH Terms] OR “placebo*”[TIAB] OR “random*”[TIAB])

The search was limited to randomised clinical trials. No language restriction was applied. We checked the reference lists of the reviewed articles and original studies identified by the electronic search for other potentially eligible articles.

Trial registers were also checked for unpublished studies.

### Study selection

One reviewer searched the literature and assessed the quality of trials using a standardized approach and a pre-specified protocol.

### Data collection and risk of bias assessment

One reviewer extracted data from the selected trials, and two reviewers (ED, LH) checked these data for accuracy. For each trial, a standard data extraction method was used to record data on the participants’ characteristics; treatment regimens; number of participants by group; primary outcome, defined as change in best corrected visual acuity (BCVA) at 1 year; anatomical efficacy parameters at baseline and achieved at the end of the follow-up (i.e., retinal thickness at fovea, intraretinal or subretinal fluid on OCT, and dye leakage on angiogram); and nature and number of serious adverse events.

The criteria used for quality assessment were sequence generation of allocation; allocation concealment; masking of participants, staff, and outcome assessors; and other sources of bias, as recommended by the Cochrane Collaboration [7]. Studies with high or unclear risk of bias for any of the first three components were classified as low quality.

### Data synthesis and analysis

The results were pooled using a random-effect model, using the odds ratio (OR) to summarize dichotomous results and the weighted mean difference (MD) to summarize continuous results, along with their 95 % confidence intervals (CI).

Statistical heterogeneity was quantified using the I^2^ statistic [9], which approximates the percentage of the total variation (within and between studies) that is due to between-study variation. In the absence of heterogeneity, fixed and random-effects models yield the same results.

Potential for publication bias was explored by visually inspecting a funnel plot of the treatment effect versus standard error and Egger’s test.

When needed, sensitivity analysis could be performed to determine whether some decisions had a major effect on the results of the review.

The meta-analyses were performed using R 2.15.1 (http://www.r-project.org) with the metafor package.

## Results

### Characteristics of included studies

#### Selection of studies

Of the 51 citations retrieved from the literature search and the trial registers search, 10 studies were eligible. Three were excluded as the results were not available: the Bevacizumab Versus Ranibizumab in Age Related Macular Degeneration AxL-2009 trial (NCT01014468, status unknown); the Prevention of Vision loss in Patients with AMD by Intravitreal Injection of Bevacizumab and Ranibizumab (VIBERA, NCT00559715, status unknown); and the Lucentis Compared to Avastin Study (LUCAS, NCT01127360, completed). For one study, results were presented at a meeting, but no publications were retrieved (the Comparison of Bevacizumab and Ranibizumab in Exudative Age-Related Macular Degeneration study, BRAMD, NTR1704; 13th EURETINA Congress, 26–29 September 2013, Hamburg).

One trial was excluded [10, 11] due to the availability of two papers for this same trial with large discrepancies between the two papers regarding data and results (notably the sample size), which meant it was impossible to choose between these two different versions.

**Fig. 1:**
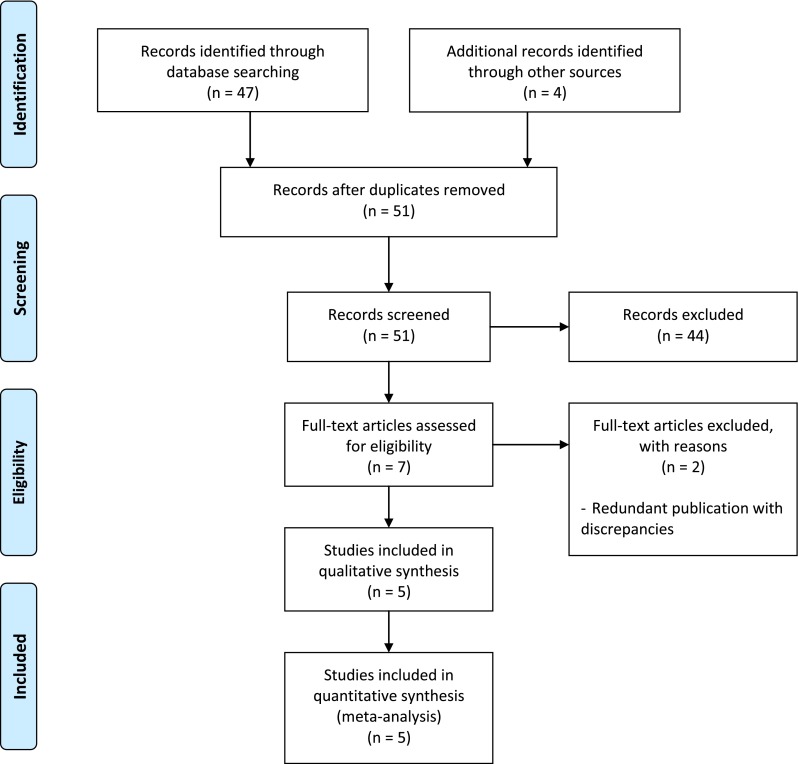
Study identification and selection flowchart

Finally, five studies were included (Fig. 1): Subramanian et al. [12]; the CATT study [2]; the Alternative Treatments to Inhibit VEGF in Age-Related Choroidal Neovascularization (IVAN) study [3]; the Multicentre Anti-VEGF Trial in Austria (MANTA) [5]; and the French study group Avastin versus Lucentis for Neovascular AMD (GEFAL) [4].

#### Methodology of the studies

Overall, two treatment regimens were identified: monthly intravitreal injections, and as-needed (Table 1). CATT presented separate results of bevacizumab versus ranibizumab (i) with monthly intravitreal injections and (ii) with an as-needed regimen; therefore, it was considered as two sets of data. Finally, six sets were included in the quantitative analysis.

**Table 1:** Characteristics of the studies included in the quantitative meta-analysis

Study	Setting	Location	Treatment regimen	Number of patients in the considered population for safety (bevacizumab/ranibizumab)	Analysis Population for primary outcome	Number of patients in the considered population for primary outcome (bevacizumab/ranibizumab)	Number of randomised patients (bevacizumab/ranibizumab)
GEFAL, 2013^4^	Multi-centre	France	As-needed	255/246	*Per protocol*	191/183	246/239
MANTA, 2013^5^	Multi-centre	Austria	As-needed	321 overall	ITT	154/163*	154/163
IVAN, 2012^3^	Multi-centre	England	As-needed and Monthly	305/323	ITT	251/269	296/314
CATT (monthly), 2011^2^	Multi-centre	USA	Monthly	1,208 overall	ITT	265/284	286/301
CATT (as needed), 2011^2^	Multi-centre	USA	As-needed		ITT	271/285	300/298
Subramanian et al., 2010^12^	Single centre	USA	As-needed	20/8	Available patients	15/7	20/8

*ITT* intention to treat

^a^Specific data were not available to be included in the meta-analysis

The primary outcome for all trials was defined as change in best corrected visual acuity (BCVA) at 1 year. Three trials were primarily designed as non-inferiority trials (CATT, IVAN, and GEFAL); the non-inferiority margins were −5, −3.5, and −5, respectively. The two others were considered as superiority trials (expected difference between treatments of seven letters for MANTA and no hypothesis defined in Subramanian et al.).

In the GEFAL and MANTA studies, participants, investigators, and outcome assessors were masked. One trial was described as single-masked (CATT), but for the between-treatments comparison, the investigator and assessor were masked. For the IVAN trial, 98.6 % of the participants and 98.7 % ophthalmologists were masked at the 12-month visit. The Subramanian et al. study was described as double-masked, with no further details reported.

Three trials (CATT, IVAN, and GEFAL) have reported adverse events according to the Medical Dictionary for Regulatory Activities (MedDRA) system.

Selective outcome bias was low in all five trials regarding visual acuity endpoints and serious adverse events (only one trial did not provide adverse events in detail [12]).

### Data analysis and synthesis

#### Patient characteristics

Overall, 2,686 patients were randomised to one of the two drugs (Table 1). The baseline characteristics of patients are presented in Table 2.

**Table 2:** Baseline characteristics of patients

	GEFAL^4^		MANTA^5^		IVAN^3^		CATT (monthly)^2^		CATT (as-needed)^2^		Subramanian et al.^12^	
	Bevacizumab	Ranibizumab	Bevacizumab	Ranibizumab	Bevacizumab	Ranibizumab	Bevacizumab	Ranibizumab	Bevacizumab	Ranibizumab	Bevacizumab	Ranibizumab
	*n* = 191	*n* = 183	*n* = 154	*n* = 163	*n* = 296	*n* = 314	*n* = 286	*n* = 301	*n* = 300	*n* = 298	*n* = 15	*n* = 7
Age (years)	79.6 (6.9)	78.7 (7.3)	76.7 (7.8)	77.6 (8.1)	77.7 (7.2)	77.8 (7.6)	80.1 (7.3)	79.2 (7.4)	79.3 (7.6)	78.4 (7.8)	78	80
Male/Female [n]	72/119	54/129	56/98	59/104	115/181	129/185	106/180	118/183	116/184	113/185	15/0	6/1
Visual acuity (ETDRS letters)	54.6 (14.1)	55.8 (14.0)	57.0 (13.0)	56.4 (13.5)	61.1 (15.6)	61.8 (15.0)	60.2 (13.1)	60.1 (14.3)	60.4 (13.4)	61.5 (13.2)	34.9 (14.5)	32.7 (20.9)
Retinal thickness at fovea* (μm)	359.21 (120.72)	354.75 (109.90)	374.6 (8.4)	365.0 (8.1)	264 (131)	271 (129)	254 (121)	251 (122)	252 (115)	247 (122)	NA	NA
Intraretinal or subretinal fluid on OCT [n (%)]	181 (94.8)	173 (94.5)	NA	NA	154 (56)	154 (53)	NA	NA	NA	NA	NA	NA

Results are mean (SD) unless otherwise specified

OCT, optical coherence tomography. NA, not available

^a^Defined as (i) central subfield macular thickness in GEFAL and MANTA; and (ii) retinal thickness plus subfoveal-fluid in IVAN and CATT

#### Functional endpoint (visual acuity)

**Fig. 2:**
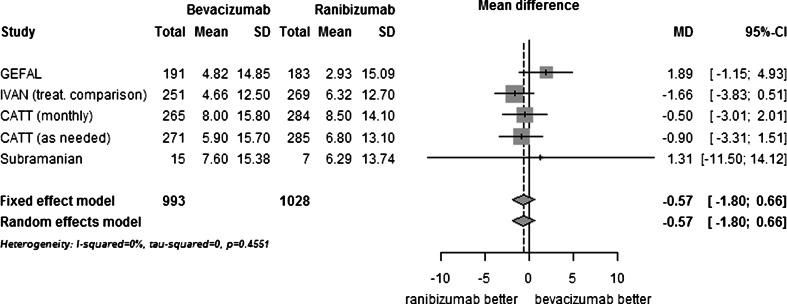
Comparison of bevacizumab with ranibizumab for mean change in visual acuity (ETDRS letters) at 1 year. CI, confidence interval; MD, mean difference; SD, standard deviation

The analysis population for the primary outcome consisted of 993 patients for bevacizumab and 1,028 for ranibizumab (MANTA did not provide the mean change in BCVA in each group and was, therefore, not included). The difference between bevacizumab and ranibizumab in terms of mean change in BCVA at 1 year was not significant (MD −0.57 letters, −1.80 to 0.66, *p* = 0.37, I^2^ = 0 %; Fig. 2). The difference was not significant for any other visual acuity parameters.

#### Anatomical endpoints

**Fig. 3:**
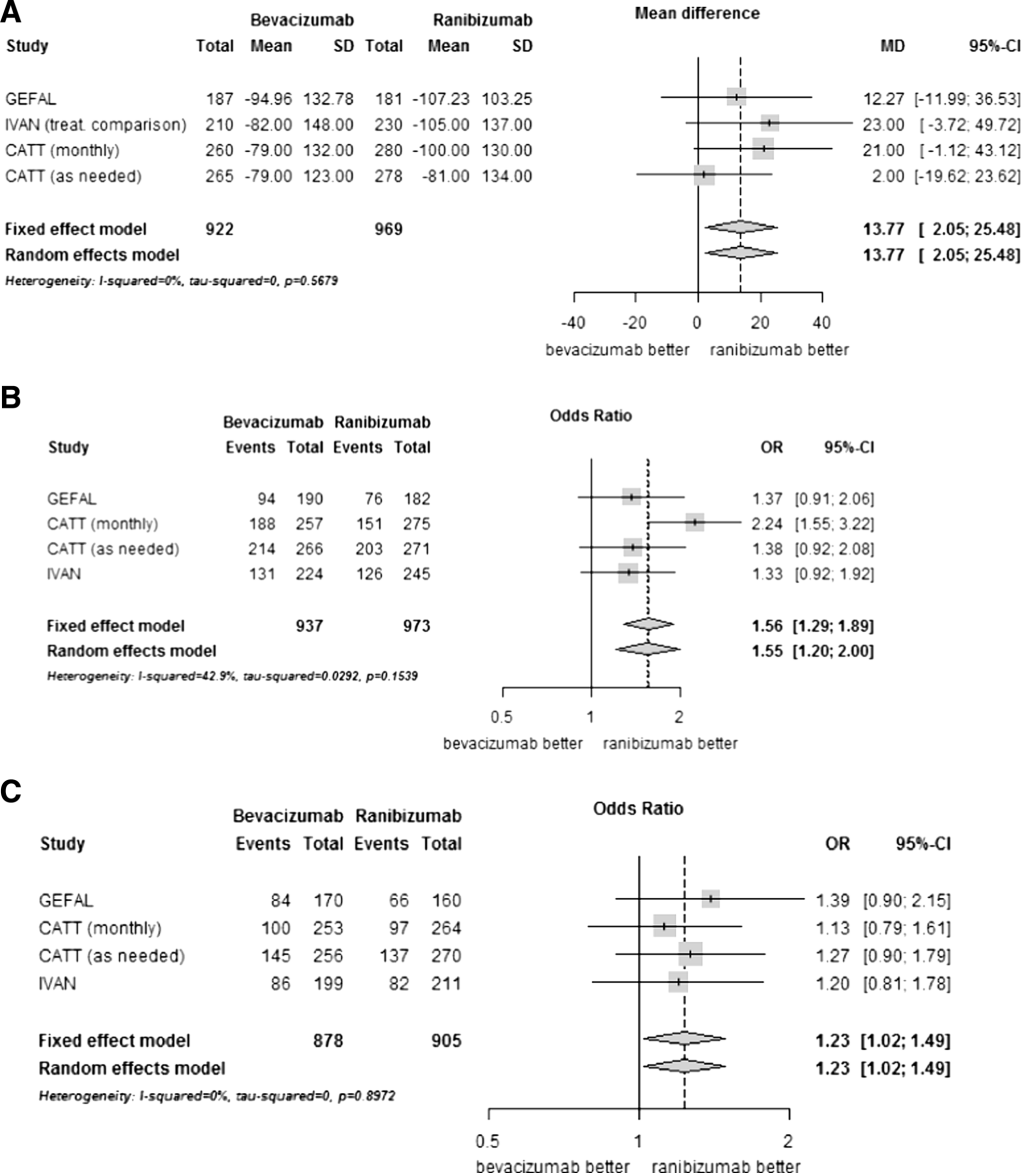
Comparison of bevacizumab with ranibizumab for anatomical results. Panel **a**, Change in retinal thickness at fovea at 1 year (microns); Panel **b**, Presence of intraretinal or subretinal fluid on optical coherence tomography at 1 year; Panel **c**, Presence of dye leakage on angiogram at 1 year. CI, confidence interval; MD, mean difference; OR, Odds ratio; SD, standard deviation

Overall, ranibizumab was associated with better anatomical outcomes (Fig. 3) in terms of change in retinal thickness at fovea at 12 months (MD 13.77, 2.05 to 25.48, *p* = 0.02), the presence of intraretinal or subretinal fluid on OCT (OR 1.56, 1.29 to 1.89, *p* < 0.01) and dye leakage on angiogram (OR 1.23, 1.02 to 1.49, *p* = 0.03).

#### Adverse events

The adverse events are presented in Table 3.

**Table 3:** Safety results

Endpoints	Number of study sets	I^2^† (%)	Fixed effect		Random effect	
			OR [95 % CI]	p value	OR [95 % CI]	p value
Systemic* serious adverse events	6	0	1.34 [1.08; 1.66]	0.01	1.34 [1.08; 1.66]	0.01
Gastrointestinal disorder	5	42	2.23 [1.09; 4.57]	0.03	2.19 [0.77; 6.21]	0.14
Infection	5	0	1.61 [0.99; 2.64]	0.06	1.61 [0.98; 2.64]	0.06
Nervous system disorder	5	7	1.07 [0.61; 1.87]	0.82	1.04 [0.55; 1.95]	0.91
Benign or malignant neoplasm	5	0	0.97 [0.52;1.81]	0.93	0.93 [0.49; 1.77]	0.83
Cardiac disorder	4	15	1.06 [0.66; 1.69]	0.82	1.04 [0.60; 1.78]	0.89
Myocardial infraction	5	0	0.83 [0.32; 2.10]	0.69	0.85 [0.32; 2.24]	0.74
Stroke	6	0	0.67 [0.23; 1.97]	0.47	0.76 [0.23; 2.46]	0.65
APTC arterial thromboembolic events	5	0	0.82 [0.42; 1.59]	0.55	0.89 [0.44; 1.80]	0.76
Venous thrombotic event	4	0	2.78 [0.82; 9.45]	0.10	2.38 [0.57; 9.84]	0.23
Death from any cause	6	0	1.32 [0.74; 2.36]	0.35	1.30 [0.71; 2.36]	0.39
Endophthalmitis	4	4	1.33 [0.33; 5.37]	0.69	1.37 [0.29; 6.53]	0.69
Serious non-ocular haemorrhage	2	0	3.77 [0.62; 22.90]	0.15	3.36 [0.52; 21.78]	0.20
Ocular Serious adverse events	5	57	1.78 [0.87; 3.63]	0.11	1.62 [0.49; 5.39]	0.43

*APTC* Antiplatelet trialist collaboration, *CI* confidence interval, *OR* odds ratio

*Excluding ocular events

†Statistical heterogeneity

**Fig. 4:**
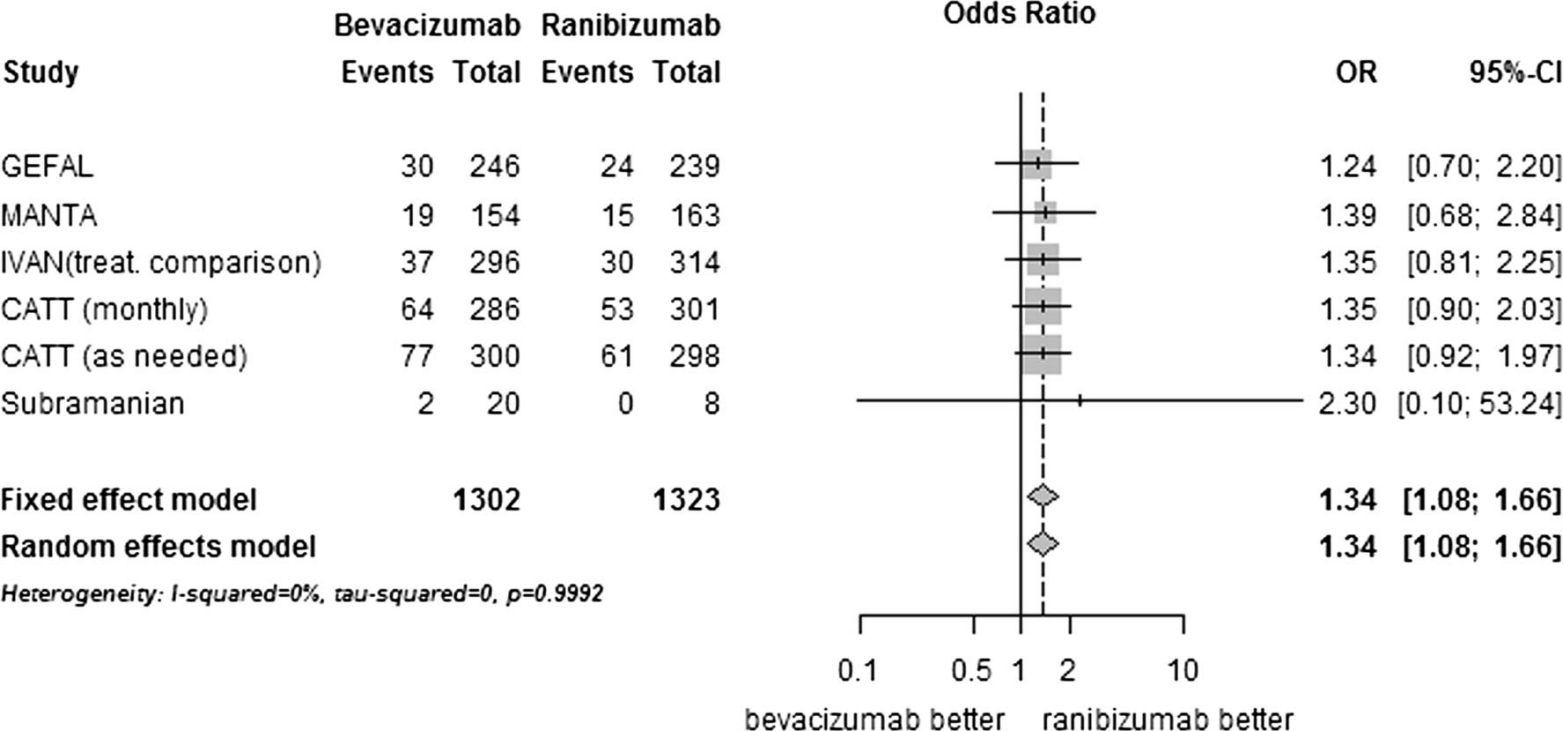
Comparison of bevacizumab with ranibizumab for systemic serious adverse events at 1 year. CI, confidence interval; MD, mean difference; OR, odds ratio; SD, standard deviation

Systemic (i.e. excluding ocular events) adverse event rates were available for all the five trials. Bevacizumab was associated with a 34 % increase in the risk of experiencing at least one serious systemic adverse event (OR 1.34, 1.08 to 1.66, *p* = 0.01; Fig. 4) compared with ranibizumab, with no heterogeneity detected (I^2^ = 0 %). No evidence of publication bias was suggested for this endpoint by visual inspection of funnel plots or by Egger’s test (two-tailed p-value of 0.17). Among the serious systemic adverse events taken individually, none were both consistently and significantly increased (Table 3). Infection was associated with a non-significant increase that was relatively consistent across the trials. An increase in gastrointestinal disorders was found with the fixed effect model, but not with the random effect model, perhaps due to heterogeneity.

A total of 33 ocular serious adverse events were reported in the trials. They were not found to be different between bevacizumab and ranibizumab (Table 3). Endophthalmitis were reported in two trials (CATT, GEFAL) and bevacizumab was not associated with a significant difference. Only one trial reported a case of uveitis (IVAN).

#### Sensitivity analysis

We tested the robustness of our analyses by performing sensitivity analyses excluding the CATT study (largest trial). When excluding data from the two sets of CATT, the systemic adverse effects were not statistically different between the groups (OR 1.33, 0.95 to 1.86, *p* = 0.09). We also performed analyses excluding alternatively CATT monthly, CATT as needed, GEFAL, and IVAN, thus ensuring similar sample size in each comparison. Only analyses including one of the CATT dataset were found significant.

## Discussion

This meta-analysis, updated with the inclusion of 2013 data from the MANTA and the GEFAL studies, did not show any difference between bevacizumab and ranibizumab in terms of the change in BCVA at 1 year in neovascular AMD.

The mean difference was −0.57 letters with a lower limit in the 95 % confidence interval of −1.80 letters. This lower bound is above all the non-inferiority margins chosen in the non-inferiority trials ( −3.5 to −5). These results complete those of the previous meta-analysis and support the functional non-inferiority of bevacizumab over ranibizumab.

This meta-analysis also confirmed the trend observed in all head-to-head studies: better anatomical results for ranibizumab, with decrease in retinal thickness at fovea, fluid, and dye leakage at 1 year. Results for retinal thickness at fovea should be considered with caution since the definitions varied between studies. The results for fluid and dye leakage have never previously been studied in previous meta-analyses. Further studies with longer follow-up periods would be required to confirm if the improvement in anatomical outcomes observed with ranibizumab is maintained over time, thus impacting on visual function and quality of life.

Concerning safety, the combined results showed a significant increase in systemic serious adverse events of more than 30 % with bevacizumab compared with ranibizumab. The two previous meta-analyses had already reported this increase, with relative risks for systemic adverse events of 1.35 (95 % CI 1.05 to 1.72, 1,795 participants) [3] and 1.3 (95 % CI 1.0 to 1.7, cumulative data from CATT publication) [6] at 1 year.

The excess risk of experiencing at least one systemic adverse event observed with bevacizumab could be surprising for a drug administered locally. Three studies demonstrated that the VEGF plasma level was significantly lower with intravitreal bevacizumab than with intravitreal ranibizumab [3, 13, 14]. VEGF is fundamental for numerous physiological extraocular functions, but the link between a lower VEGF serum concentration and higher serious adverse events has not yet been proven. Understanding the mechanism by which the systemic diffusion and action of bevacizumab occur after intravitreal injections, and identifying the potential risk factors associated with this mechanism, could contribute to identifying which patients should be treated with caution when undergoing bevacizumab intravitreal treatment.

However, serious systemic adverse events would require more advanced analysis. Indeed, our sensitivity analyses tended to lower the effect. Although the treatment effect size was similar across studies, the rate of events differed between CATT and other studies (20 % of events versus 11 %), thus suggesting that there might be some heterogeneity.

None of the side effects of bevacizumab seen after systemic use in the early wet AMD studies [15], especially hypertension, or the cancer studies could be confirmed in any of the head-to-head wet AMD-studies. However, none of the five studies included in this meta-analysis was designed or powered to assess safety concerns.

Moreover, as no serious adverse event taken individually was significantly increased in our analysis that included 2,625 patients (taking into account the heterogeneity), it is not possible to explain clearly this overall excess of systemic adverse events. A safety signal could be suspected for infections. This signal was found consistently in all the trials included in this meta-analysis, but was not statistically significant. Given the safety profile of systemic administrations of bevacizumab, the recommendation could be to monitor patients closely for infection. Especially, cases of epidemic endophthalmitis have been previously reported, and might be increased due to repackaging of vials of bevacizumab in single syringes in the absence of a commercially specific packaging for intravitreal administration. For the preparation of off-label bevacizumab, one could recommend either the use of one vial per patient such as in GEFAL, or, from a money-saving perspective, an industrial repackaging in an aseptic filing facility such as in CATT.

This meta-analysis is the most comprehensive review of literature assessing the relative efficacy and safety of bevacizumab and ranibizumab in neovascular AMD.

Despite the absence of statistical heterogeneity for most of the outcomes studied, the selected studies were clinically heterogeneous in terms of treatment regimens. Moreover, their statistical perspectives (non-inferiority or superiority) and populations (intention-to-treat or *per protocol* sets) also differed. Finally, the final BCVA value used in the analyses varied, as some trials performed imputations for missing data at 1 year.

However, the objective of the meta-analysis was to analyse the effect of the treatment independently of the treatment regimen; the duration and doses of the drugs were identical for most of the studies. Furthermore, there was no random error for mean change in BCVA at 1 year (primary efficacy outcome) as no heterogeneity was found between the results of the trials.

The analysis of safety events is also prone to several biases as the data varied in each study in terms of quality, incidence, severity, and adjudication. In RCTs, all SAEs must be specifically investigated and recorded regardless of the imputability with the drug. The reporting may also be influenced by the expectations of the investigators, sponsors, and patients. However, the definitions for safety outcomes were based on the MedDRA system for three out of five selected trials, representing 2,280 of the 2,625 patients in the safety population. Despite the exploratory nature of the safety analysis, no heterogeneity was observed, which reinforces our results on systemic serious adverse events as it means they cannot be imputed to artifactual data.

A further limitation to this review is that at least four more trials comparing bevacizumab and ranibizumab for visual acuity (primary outcome measured at 1 year) in AMD have been identified, but at the time of our search, the results were not published.

This meta-analysis found sufficient evidence to conclude that bevacizumab is associated with similar effects on visual acuity compared with ranibizumab. It also showed that bevacizumab may be associated with an excessive risk of systemic serious adverse events. However, the current available data do not show which types of adverse events occur more frequently. In practice, bevacizumab for neovascular AMD should be used under a risk management plan. The main explanation for the current use of bevacizumab is economic, reinforced by an equivalent functional efficiency with ranibizumab, but this should be balanced against the poorer anatomical results and a suspected higher rate of serious systemic adverse events than ranibizumab at 1 year.
